# Perforation of the Brachial Artery During Percutaneous Lower Extremity Angioplasty via the Brachial Artery Approach Resulting in Difficulties in Balloon Catheter Removal: A Case Report

**DOI:** 10.7759/cureus.48590

**Published:** 2023-11-09

**Authors:** Yusuke Yoshishige, Katsuro Kashima, Kenichi Arata, Masaomi Ooi, Kazuyo Kawabata

**Affiliations:** 1 Cardiology, National Hospital Organization Ibusuki Medical Center, Ibusuki, JPN; 2 Cardiovascular Surgery, Kagoshima City Hospital, Kagoshima, JPN

**Keywords:** case report, endovascular surgical repair, vascular perforation, transbrachial, endovascular intervention

## Abstract

Percutaneous endovascular treatment of peripheral vascular disease with small-caliber short sheaths may lead to device removal difficulties. A 50-year-old woman on hemodialysis underwent endovascular intervention for right common femoral artery stenosis, via the right brachial artery. A 4-Fr short sheath was used for the procedure owing to a previous hematoma at the puncture site. However, the balloon catheter could not cross the calcified lesion and was difficult to remove. A microcatheter was inserted and withdrawn, but the guidewire was kinked and could not be retrieved. Surgical retrieval of the guidewire and balloon catheter was performed. The kinked guidewire and microcatheter had migrated outside the vessel. In peripheral vascular intervention, the use of a long sheath in the brachial artery approach is important. Forcible removal of a difficult-to-remove catheter may cause further vascular damage. Therefore, it is essential to stop immediately and consider surgical treatment.

## Introduction

The access site for percutaneous vascular intervention is determined on the basis of an individual patient’s clinical situation. The femoral, brachial, and radial arteries each have associated benefits and risks [1]. Radial artery access has become the preferred approach for percutaneous vascular intervention because of its lower risk of vascular and bleeding complications [2]. However, in hemodialysis patients, access is limited to the brachial or femoral artery owing to the presence of vascular shunts.

In transbrachial vascular interventions, access site complications are more common compared with femoral or radial artery approaches [3]. As the risk of these complications increases with sheath size, small-diameter sheaths are often used to reduce the risk of bleeding [4]. However, it is important to understand the risks of using short, small-diameter sheaths and the importance of using long sheaths, which are commonly used in percutaneous vascular interventions [5].

We present the details of a patient who underwent percutaneous vascular intervention with a short, small-diameter sheath. The device could not cross the lesion, and the guidewire became kinked during catheter manipulation, making the device difficult to remove.

This article was previously posted to the Research Square preprint server on October 10, 2023.

## Case presentation

**Figure 1:**
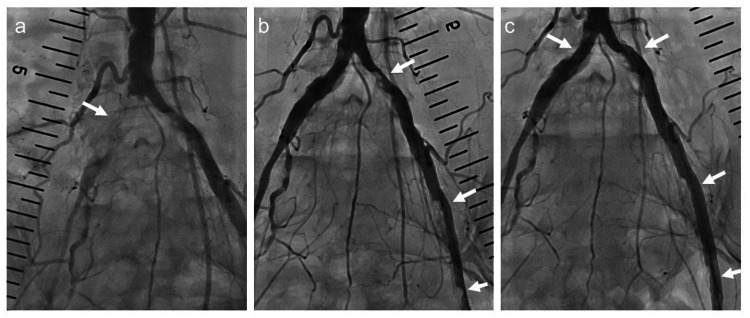
Lower extremity arteriography findings during the previous hospitalization. (a) The right common iliac artery (CIA) is completely occluded (arrow). (b) The left CIA and left external iliac artery show stenosis (arrows). (c) No significant stenotic lesions are visible at the stent placement sites of both iliac arteries (arrows) associated with the previous treatment.

A 50-year-old woman with chronic kidney disease on hemodialysis presented with rest pain in the right lower extremity. Her serum electrolytes were kept within normal range by regular hemodialysis. She had a history of right common iliac artery (CIA) occlusion and previously underwent percutaneous vascular intervention with a right brachial and right femoral artery approach and stent placement in the right CIA (Figures 1a, 1b). She had a minor hematoma at the right brachial artery puncture site and post-operative pain for several days. Subsequently, she underwent percutaneous endovascular intervention with a left common femoral artery (CFA) approach for left CIA and left external iliac artery stenosis (Figures 1b, 1c).

**Figure 2:**
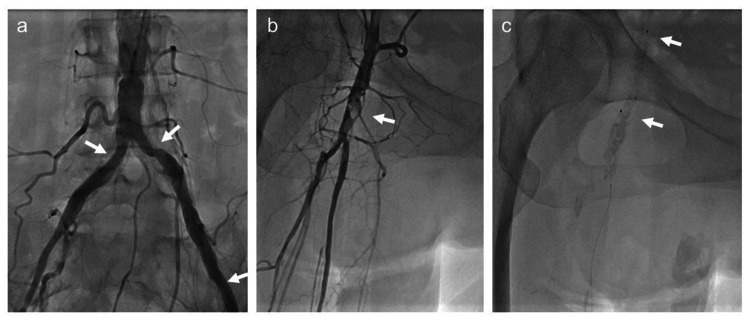
Lower extremity arteriography findings during the present hospitalization. (a) No significant stenotic lesions are visible at the stent placement sites of both iliac arteries (arrows). (b) The right common femoral artery shows a severe stenotic lesion with significant calcification (arrow). (c) The balloon catheter could not pass through the stenosis (arrows).

At the current admission, she presented with a low ankle-brachial index in the affected leg (left: 0.71, right: 0.25) and a calcified, severely stenotic lesion in the right CFA (Figure 2b). Owing to the previous vascular intervention complication, a 4-Fr short sheath was inserted into the right brachial artery for angiography. Treatment was initiated with the 4-Fr short sheath, without changing to a 6-Fr long sheath. However, the monorail balloon catheter could not pass beyond the lesion (Figure 2c) and became difficult to remove when forcibly advanced.

**Figure 3:**
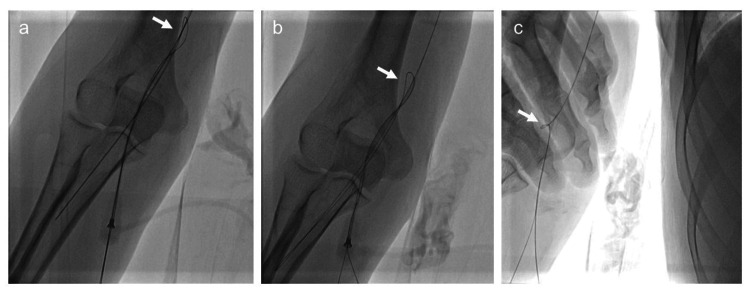
Guidewire kinking during the therapy. (a) Balloon catheter removal was difficult owing to guidewire kinking (arrow). (b) The guidewire was twisted above the sheath at the right brachial artery puncture site (arrow) during forceful manipulation of the balloon catheter. (c) Straightening the guidewire was attempted, but the kink was difficult to eliminate (arrow).

The catheter was retracted to the point of resistance but could not be retrieved distal to the right subclavian artery. Additionally, the guidewire could not be straightened in the brachial artery (Figures 3a, 3b). A microcatheter was inserted, but the guidewire remained kinked (Figure 3c), and the balloon catheter could not be removed.

**Figure 4:**
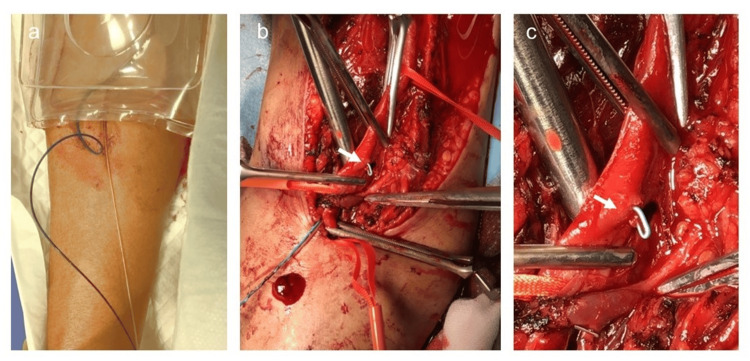
Perforation of the right brachial artery. (a) Microcatheter insertion failed to achieve catheter removal; therefore, the sheath was removed, and hemostasis was achieved with a hemostasis device (Zemex Hemostatic System Tometakun^(R)^, Zeon Medical Inc. Japan); (b) Under surgical exploration, a vascular injury was found 3 cm proximal to the puncture site. The guidewire and microcatheter were located outside the vessel at the site of the injury (arrow). (c) Perforation of the brachial artery is visible (arrow).

After the sheath was removed, and hemostasis was achieved using a hemostatic device (Zemex Hemostatic System Tometakun^(R)^, Zeon Medical Inc. Japan) (Figure 4a), the patient was urgently transferred to the cardiovascular surgery department at Kagoshima City Hospital. A vascular injury was found 3 cm from the puncture site during exploration of the brachial artery. The guidewire and microcatheter had kinked and migrated out of the vessel at the same location (Figures 4b, 4c). Under fluoroscopy, the segment of the vessel affected by the device migration was excised, with the device, followed by vessel repair and surgery completion.

The patient’s post-operative right upper arm pain resolved within 3 days of surgery. Approximately 2 months later, she underwent a right CFA endarterectomy, which resulted in improved peripheral circulation.

## Discussion

In patients on hemodialysis, vascular access for endovascular treatment of lower extremity peripheral artery disease is limited to the contralateral brachial or femoral artery owing to the presence of a forearm arteriovenous shunt. In the present case, percutaneous treatment was performed for right CFA disease. However, owing to the presence of a stent extending from the right CIA to the proximal descending aorta, the contralateral femoral artery was not accessible, and the right brachial artery was the only accessible vessel. Additionally, the patient had a previous hematoma at the site of the right brachial artery puncture. To prevent recurrence, the smallest possible diameter sheath was used in the current vascular intervention.

The incidence of brachial artery puncture-related complications during percutaneous vascular intervention is reported to be 6.5%, with thrombosis and pseudoaneurysm being the most common complications. Of these cases, 65% required surgical intervention [6]. Additionally, the use of larger sheaths is associated with an increased risk of complications, including bleeding [4]. Therefore, small-diameter sheaths are often used to reduce the risk of bleeding complications. In patients with tortuous iliac arteries, the use of long interventional sheaths improves access to the lesion [5].

The present patient underwent vascular intervention with a small-diameter short sheath to avoid bleeding complications. As a result, the balloon catheter was difficult to remove. Regarding the cause of the catheter removal difficulty, the guidewire may have kinked and curled in the central portion of the guidewire lumen outlet of the balloon catheter when the balloon catheter was forcefully advanced. The guidewire may have twisted during forceful manipulation of the balloon catheter, which caused the vessel wall perforation when the guidewire was retracted to straighten it [7].

The guidewire kinking could have been prevented with the use of a long sheath or by carefully handling the catheter and guidewire. In cases where the catheter is kinked and cannot be removed, there is a technique for catheter kink management via “swallowing” a long sheath [2]. However, in cases where the vessel has already been perforated, forceful catheter removal may damage the vessel further. Therefore, it is important to stop the interventional procedure immediately and consider surgical removal.

Peripheral arteries in hemodialysis patients often have severe calcification, which is associated with an increased risk of cardiovascular events and a poor prognosis [8,9]. Therefore, percutaneous intervention for peripheral arteries in hemodialysis patients should be considered carefully, especially the risk of difficult catheter removal when using small-bore short sheaths. This case stresses the importance of using long sheaths, which is a basic strategy in brachial access.

## Conclusions

Long sheaths must be used in brachial access for percutaneous vascular interventions because small-bore short sheaths can lead to difficult device removal. Perforation of the vessel may be suspected if catheter removal is difficult. Forcible removal may cause further damage to the vessel and is therefore contraindicated. Early surgical intervention is recommended in such cases.
